# CANGS DB: a stand-alone web-based database tool for processing, managing and analyzing 454 data in biodiversity studies

**DOI:** 10.1186/1756-0500-4-227

**Published:** 2011-06-30

**Authors:** Ram Vinay Pandey, Viola Nolte, Jens Boenigk, Christian Schlötterer

**Affiliations:** 1 Institut für Populationsgenetik, Veterinärmedizinische Universität Wien, Veterinärplatz 1, Vienna, Austria; 2 Allgemeine Botanik, Universität Duisburg-Essen, D-45117, Essen Germany

## Abstract

**Background:**

Next generation sequencing (NGS) is widely used in metagenomic and transcriptomic analyses in biodiversity. The ease of data generation provided by NGS platforms has allowed researchers to perform these analyses on their particular study systems. In particular the 454 platform has become the preferred choice for PCR amplicon based biodiversity surveys because it generates the longest sequence reads. Nevertheless, the handling and organization of massive amounts of sequencing data poses a major problem for the research community, particularly when multiple researchers are involved in data acquisition and analysis. An integrated and user-friendly tool, which performs quality control, read trimming, PCR primer removal, and data organization is desperately needed, therefore, to make data interpretation fast and manageable.

**Findings:**

We developed CANGS DB (Cleaning and Analyzing Next Generation Sequences DataBase) a flexible, stand alone and user-friendly integrated database tool. CANGS DB is specifically designed to organize and manage the massive amount of sequencing data arising from various NGS projects. CANGS DB also provides an intuitive user interface for sequence trimming and quality control, taxonomy analysis and rarefaction analysis. Our database tool can be easily adapted to handle multiple sequencing projects in parallel with different sample information, amplicon sizes, primer sequences, and quality thresholds, which makes this software especially useful for non-bioinformaticians. Furthermore, CANGS DB is especially suited for projects where multiple users need to access the data. CANGS DB is available at http://code.google.com/p/cangsdb/.

**Conclusion:**

CANGS DB provides a simple and user-friendly solution to process, store and analyze 454 sequencing data. Being a local database that is accessible through a user-friendly interface, CANGS DB provides the perfect tool for collaborative amplicon based biodiversity surveys without requiring prior bioinformatics skills.

## Background

Next generation sequencing technologies are delivering data at a hitherto unprecedented speed and dramatically reduced costs. In addition to genome sequencing and transcriptome profiling, ultra-deep sequencing of short amplicons offers an enormous potential in clinical studies [1] and in surveys of ecological diversity [2-4]. Typical biodiversity surveys include sequences from a diverse set of samples. An effective data analysis requires the ability to link additional data, such as time of collection and ecological variables, to the sequences. Furthermore, biodiversity surveys often require sequence information on different taxonomic levels. Hence, researchers need an analytical tool that provides the flexibility to handle different PCR primers.

Until now several tools have been developed, but none of them unite all of the requirements for a comprehensive tool. In the following we briefly introduce these tools, highlight their features, and discuss missing options.

1) RDP [5] is an online tool for sequence trimming and filtering. It provides an excellent taxonomic classifier, which is, however, limited to small ribosomal subunit gene sequences from bacteria and archea. Furthermore, it provides no option to store and manage data provided by the user. MOTHUR [6] combines read trimming and filtering capabilities along with rare-faction analyses. MOTHUR is a command line software and provides many useful utility commands for biodiversity studies but it does not offer a data storage option. CANGS [7] and CANGS DB rely on MOTHUR for rarefaction analyses. VAMPS [8] provides sequence trimming, filtering of low quality reads and taxonomic path assignment using the GAST pipeline. The user can upload data for visualization and analysis of microbial population structures. The limitation of VAMPS is a rigid sequence-processing pipeline that does not allow for user-defined options (e.g.: reads are only filtered allowing for ambiguities, it is not possible to define a size range for amplicon sizes, and quality scores of the sequence reads are not accounted for). Furthermore, it is not possible to store additional data about the sequences, such as ecological variables. Finally, the user cannot retrieve data according to user-defined criteria. PANGEA [9] allows for trimming of the barcodes and groups sequences according to the barcode. PANGEA has many useful features including clustering, classification, and comparison of microbial communities. While PANGEA uses a local database for classification, it is not designed to incorporate user-generated sequences into this database. Thus, data manipulation and organization of 454 data from multiple runs is not possible.

We developed CANGS DB (http://code.google.com/p/cangsdb/) as an integrated user-friendly database tool that can be easily installed on local computers and accessed through the internet by standard browsers. It offers a flexible, customizable sequence-processing pipeline where 454 sequences can be uploaded/downloaded and data can be manipulated via a user-friendly interface. A variety of tools are available in the CANGS DB web interface for the downstream analysis of stored 454 sequencing data. CANGS DB links external information, such as details about the collection site, time of the year and environmental variables, to the sequence information. This allows the user to extract sequences according to combinations of particular variables (e.g.: all sequences obtained from water samples with a given temperature). A demo of CANGS DB is running on http://i122mc100.vu-wien.ac.at/CANGSdb/

## Construction and content

### Database and web interface development

**Figure 1:**
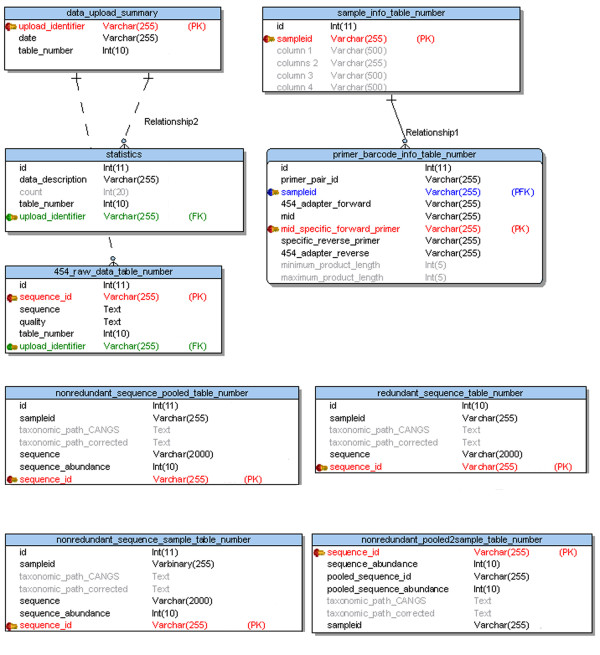
**The CANGS DB database Entity Relationship Data Model (ERDM)**. There are two static tables (data_upload_summary and statistics) and seven dynamic tables (sample_info_ table_number, primer_barcode_info_ table_number, raw_454_data_ table_number, redundant_sequence_table_number, nonredundant_sequence_pooled_ table_number, nonredundant_sequence_sample_ table_number, nonredundant_pooled2sample_ table_number), CANGS DB creates these dynamic set of tables with each new data set. In this figure the primary key (PK) is given in red color, Foreign key (FK) is given in green color and Primary+Foreign key (PFK) is given in blue color.

The CANGS DB is completely written in Perl and uses data stored in a relational database (MySQL). The relational database schema is shown in Figure 1. CANGS DB web interface is developed using the CGI.pm and runs of Apache (2.0.53) web server. The interaction between user interface and database is established by using DBI.pm and DBD::mysql.pm modules. CANGS DB can be run on Mac OS, Linux and other Unix-like systems.

Required programs are Bioperl [10], BLAST [11] for the similarity search in the taxonomy analysis tool, MAFFT [12] for pairwise distance calculation (MAFFT is used for pairwise alignment) in the rarefaction analysis tool, MOTHUR [6] for estimating the number of species (OTUs), MySQL [13] for data storage, update_blastdb.pl [14] for downloading the BLAST database on a local computer, and R [15] to plot rarefaction curves from MOTHUR output.

### Processing the raw sequence data

**Figure 2:**
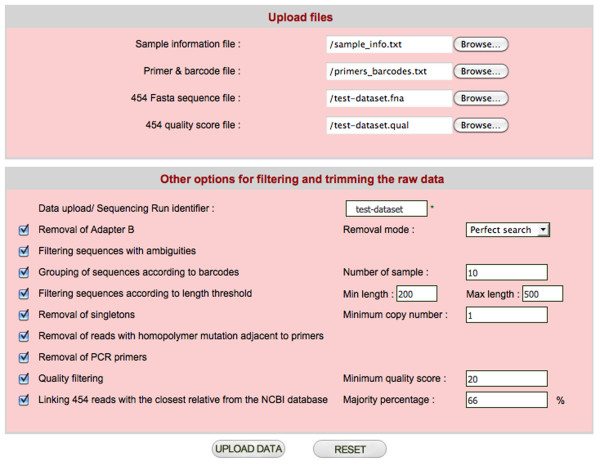
**The 454 sequence trimming and filtering interface**. Interface for trimming and quality filtering of new sequences to be added to the database.

CANGS DB uses the basic pipeline of CANGS [7], but has been modified to provide more flexibility and some additional features. CANGS DB processes raw sequences to provide the user with high quality 454 reads by trimming the adapter B, barcodes and PCR primers and filtering the low quality reads as described below. The sequence-processing pipeline of CANGS DB is highly flexible and user friendly. Each step in the pipeline can be modified on the interface (Figure 2). Moreover, CANGS DB is able to handle multiple primers of different length in the trimming step. Most importantly, filtering according to sequence length can be individually defined for each amplicon.

#### 1. Removal of adapter B

based on the sequences of adapter B, as specified in the primer & barcodes input file, the 3'- end of each read is trimmed. It is possible to process only sequences with a perfect match to adapter B. Alternatively, a pattern search that allows for imperfection in adapter B can also be used, allowing more sequences to be recovered. CANGS DB also enables trimming multiple adapter B sequences of different lengths. Poly N tails at the 3' end of the 454 reads are removed before Adapter B trimming.

#### 2. Filtering sequences with ambiguities

CANGS DB allows the removal of reads with one or more Ns (unknown bases).

#### 3. Grouping of sequences according to bar codes

CANGS DB groups sequences based on the barcodes specified in the primer & barcodes input file. Sequences with the same barcode are grouped into one category. This step is skipped when only a single sample is processed. CANGS DB is designed to allow for barcodes of different length.

#### 4. Removal of singletons

to ameliorate the problem of sequencing errors and chimeric sequences generated by jumping PCR, CANGS DB allows the user to remove low frequency variants from the data set. Sequences must be present in at least two different samples in the entire data set before trimming of the primers. This is more stringent than the former criterion implemented in CANGS [7], namely a sequence being present two or three times in the whole dataset. By conditioning on the presence in two independent data sets CANGS DB excludes chimeric sequences more efficiently, as a chimeric sequence could occur multiple times in a data set, in particular if the chimeric molecule has been generated during the early PCR steps. Note that several data sets could be combined to minimize the removal of true low frequency sequence variants.

#### 5. Filtering sequences according to length threshold

CANGS DB removes sequence reads falling outside the size range specified in the primer & barcode input file.

#### 6. Removal of PCR primers

forward and reverse PCR primers are specified in the primer & barcode input file and removed from the sequence. Only sequences with perfect identity to the specified PCR primers are processed. The 454 sequencing process preferentially generates length variants in homopolymers. As homopolymers can be as short as two bases and the target sequence is frequently not known, we developed a special procedure to recognize such sequencing errors at the end of the PCR primer as described in [7].

#### 7. Quality filtering

CANGS DB averages the quality values for each base in a read. Quality values are taken from the .qual file after the values corresponding to adapter B, bar code and primer bases have been removed in step 1, step 3 and step 6, respectively. Sequence reads with a quality value lower than the threshold specified in the user interface page will be discarded. Note that the quality filtering may result in new singletons, which remain in the data set, as the quality filtering is the last step in the analysis.

After trimming the sequence reads, CANGS DB creates a non-redundant sequence data set in order to reduce the computational burden for further analysis. In the non-redundant sequence data set each sequence variant is only represented once. Note that in this step indels are considered to be informative. Hence, two sequences differing only by an indel will be listed independently in the non-redundant data set. The frequency of each sequence in the non-redundant data set is included in the FASTA header. The output file contains non-redundant reads that are ranked by copy number in descending order.

### Taxonomic classification assignment

CANGS DB uses the CANGS [7] taxonomy analysis pipeline to assign a taxonomic path to the newly sequenced 454 reads. CANGS taxonomy analysis pipeline does not rely on a pre-curated database for either 16S or 18S sequence like RDP classifier [5], thus it can handle sequences from any genomic region.

## Utility and Discussion

### Query interface

One of the unique features of CANGS DB is its powerful query interface. The query interface was designed with the diversity of 454 data (ecological or clinical) in mind that need to be stored and queried. In case of ecological survey sequencing data, users can retrieve subsets of the sequences in the data base according to user defined variables, such as time of the year, temperature, pH, sampling location etc. In case of clinical data, the user can retrieve sequences according to tissue type, experiment date, sampling date, time, and other related information.

**Figure 3:**
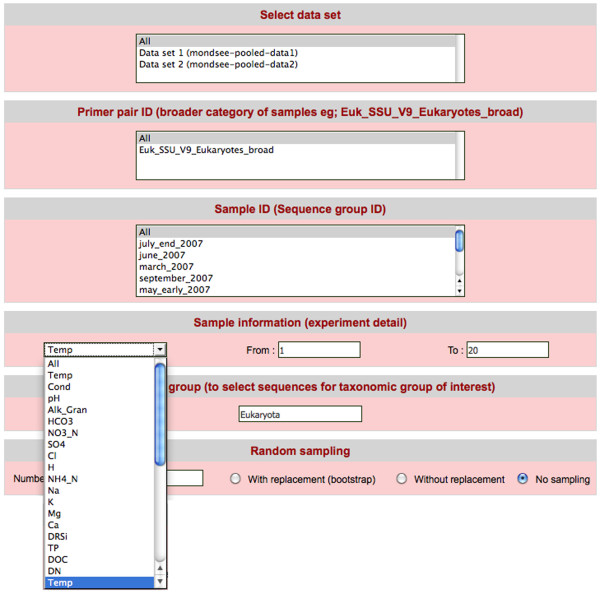
**The CANGS DB data retrieval query interface**.

Figure 3 shows how the data search could be customized:

1)	According to data sets: it is possible to select any combination of data sets loaded in the database.

2)	According to PCR primer ID

3)	According to sample ID: the use of barcodes permits the sequencing of multiple samples in one experiment. This option permits the user to restrict the search to specific sample IDs.

4)	According to external sample information: A drop down menu displays all external data categories (e.g.: temperature, pH, depth, conductivity etc). The user can then specify the desired range for each of the external data categories (e.g.: temperature range: 10-20° C)

5)	According to taxonomic group

6)	According to sample size: The user can select a sample size and obtain random samples from all selected data sets for downstream analysis. This is particularly helpful for analyses that are sensitive to different sample sizes.

The query interface is also integrated with the Blast and rarefaction interfaces so that these analyses can be performed on the subsequent query output.

### Sequence Analysis Tools

CANGS DB provides several tools for the analysis of stored data.

**Figure 4:**
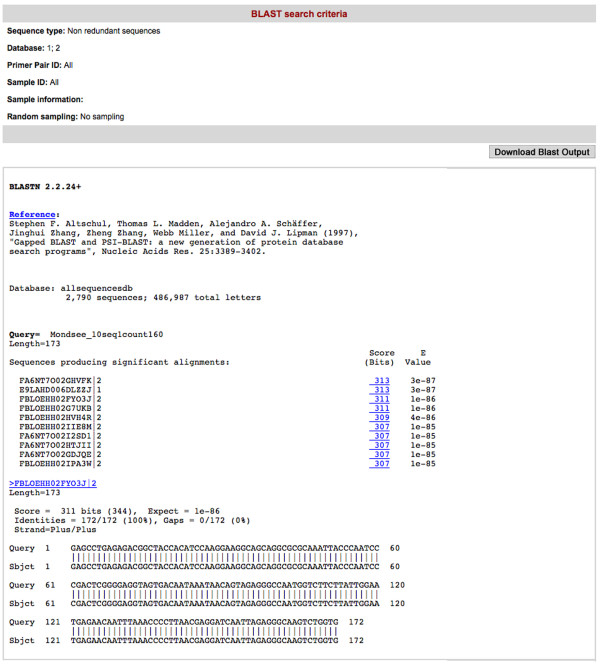
**An example output of the Blast Analysis tool**. An example output of the Blast analysis tool in CANGS DB. It shows BLAST hits for the query sequences against the selected data sets stored in CANGS DB.

**Figure 5:**
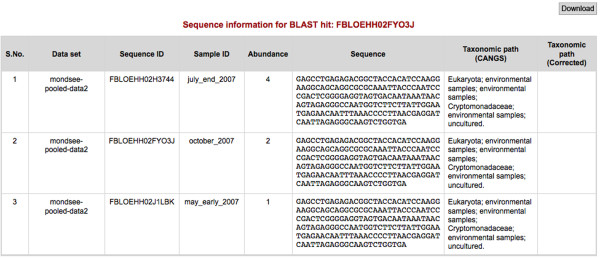
**An example output of the abundance of BLAST Hits**. An example output of the abundance of each BLAST hit in the CANGS DB. This feature provides information about the abundance and turnover of species among samples.

**Figure 6:**
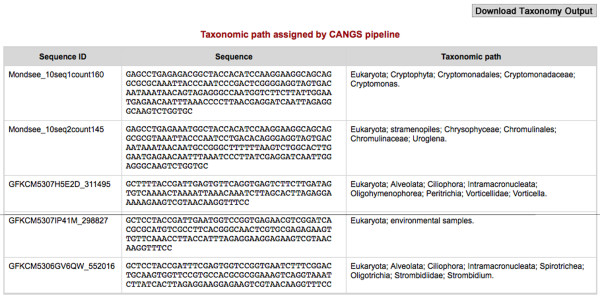
**An example output of the Taxonomy Analysis tool**. An example output of the Taxonomy Analysis from CANGS pipeline. It is a tabular output, the columns from the left to the right are 1) query sequence ID 2) query sequence 3) taxonomic path of the closest NCBI sequence.

**Figure 7:**
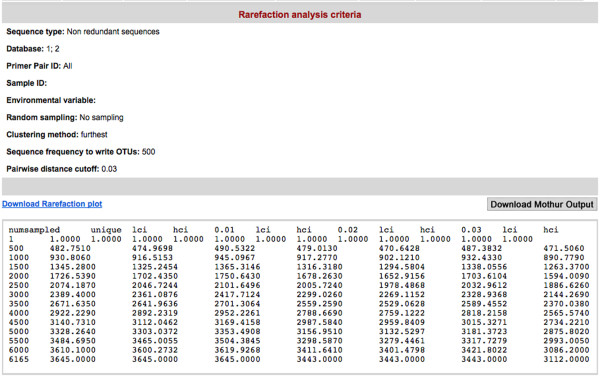
**An example output of the Rarefaction Analysis tool**. An example output of the rarefaction analysis generated by MOTHUR program.

**Figure 8:**
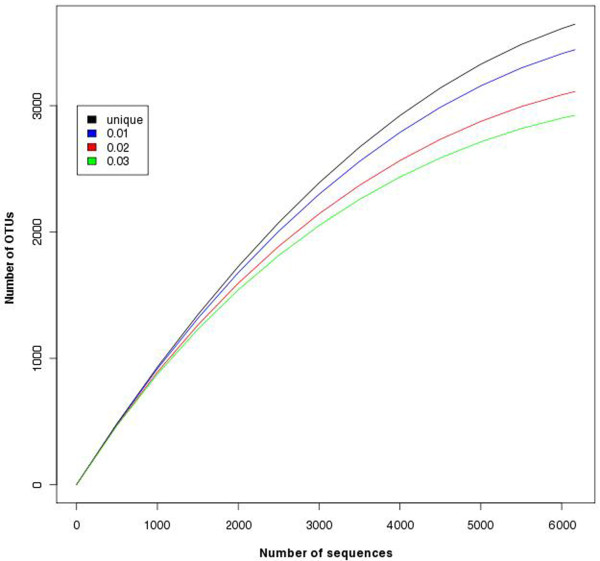
**An example output of the Rarefaction plot from Rarefaction Analysis tool**. An example output of the rarefaction plot created based on MOTHUR output.

(1) **Blast analysis tool **(Figure 4): it performs a BLAST search of any user provided query sequences against data stored in the CANGS DB MySQL database. This analysis provides the user with an estimate about the abundance of the queried sequences in the databank. Most important, by specifying the queried dataset this tool allows the user to ask specific questions (e.g.: does this sequence occur in samples collected in May?). Additionally, the user can retrieve the abundance of each hit sequence by clicking on it (Figure 5)

(2) **Taxonomy analysis **[7]: this tool classifies the query 454 reads by assessing their similarity to taxonomic entries in the NCBI database (Figure 6). This analysis requires the nucleotide preformatted BLAST database from ftp://ftp.ncbi.nih.gov/blast/db/ be installed, which is done using the perl program "*update_blastdb.pl" *[14].

(3) **Rarefaction analysis tool **[7]: this tool is used for estimating the species richness in given pooled sequences (Figure 7). CANGS DB also generates a rarefaction plot based on the MOTHUR output (Figure 8).

### Editing of sequence information

CANGS DB provides a user-friendly interface to update existing information, add new information or delete information. CANGS DB also allows users to edit the taxonomic path for any stored sequences. CANGS DB does not overwrite the edited taxonomic path; rather it keeps the edited taxonomic path in an additional column. This option is particularly helpful if multiple users work on the same data set, as it permits experts to correct the automated species assignation by CANGS DB and these changes are traceable for all users.

### Re-processing stored 454 sequences

**Figure 9:**
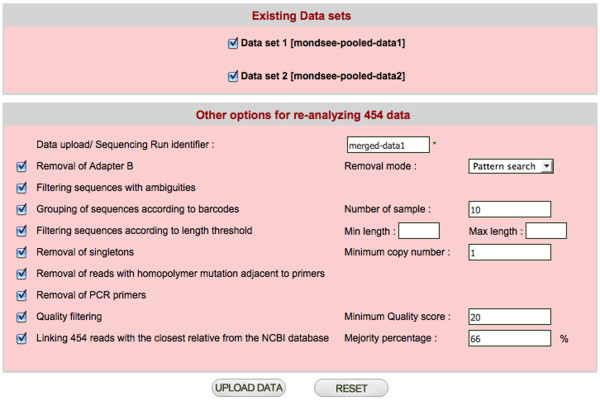
**The interface for 454-sequence re-analysis of data already up-loaded in the database**. Interface for the re-analysis of data already up-loaded in the database.

CANGS DB provides a unique feature (Figure 9), which enables users to combine raw 454 sequences uploaded into CANGS DB database into a single dataset, which can then be trimmed and filtered. This feature will be especially useful when the same samples are sequenced in different 454 runs/plates.

### Deleting dataset

CANGS DB provides option to delete any uploaded data set.

### Evaluation of the database

CANGS DB has been designed to provide maximum flexibility for the user. To demonstrate the efficiency of CANGS DB sequence processing pipeline, we processed and analysed 454 sequences previously deposited in the NCBI database [NCBI: SRA008706.2]. This data set consists of 447,909 reads from the 18S rRNA gene obtained from 10 temporal freshwater samples. Applied to our example data set, the CANGS DB sequence-processing pipeline eliminated approximately 37% of all sequences (Table 1), leaving a total of **281,003 (~63%) **sequences for downstream analyses. CANGS DB took 2.5 hours to process this data set using a Macintosh OS X version 10.6.4 with a single processor. If the user skips the removal of singletons then it takes only 20 minutes to process the same data set. When including the CANGS Taxonomic assignment pipeline the total processing time increases to 22 hours.

**Table 1:** Number of reads eliminated at different steps of the CANGS DB sequence processing pipeline

Order of steps	Steps	Total no. of sequences	No. of sequences considered	No. of sequences discarded
1	Removal of Adapter B	447,909	373,116	74,793

2	Filtering sequences with ambiguities	373,116	357,926	15,190

3	Removal of singletons	357,926	311,425	46,501

4	Grouping of sequences according to bar codes	311,425	306,042	5,383

5	Filtering sequences according to length threshold	306,042	305,884	158

6	Removal of PCR primers	305,884	282,053	23,831

7	Quality filtering	282,053	281,003	1,050

	Total Sequences	447,909	281,003	166,906

### Reproducibility

In order to increase transparency and reproducibility of the results, CANGS DB prints a log file for all processed raw data. This includes the parameter and summary statistics used for each step taken in sequence processing along with discarded sequence identities. Furthermore, stored sequences can be downloaded from CANGS DB according to the user-defined criteria.

### Future Directions

The current version of CANGS DB is only compatible for Unix operating systems; however, we plan to make it PC compatible. Additionally we will integrate more downstream analysis tools in the CANGS DB web interface.

## Conclusion

CANGS DB is a user-friendly and stand-alone database tool for processing, analyzing and managing the high throughput sequencing data from 454 amplicon resequencing projects. CANGSDB is very easy to use; it could be installed and used on any local UNIX based computer to handle individual as well as multiple sequencing projects in collaboration. It provides full-fledged flexibility with various options in raw sequence processing and analysis. CANGS DB provides a very powerful data retrieval interface, which enables researchers to retrieve information on samples, primers and barcodes from any individual data set or from a combination of data sets. It also provides an interface to update sample information and taxonomic classifications assigned by CANGS taxonomy analysis pipeline as well as delete any data set. The tool can be downloaded at http://code.google.com/p/cangsdb/.

## Availability & requirements

**Project name**: CANGS DB --Cleaning, Analyzing and Managing 454 sequences.

**Availability**: http://code.google.com/p/cangsdb/

**Operating System**: Mac OS X, Linux and any other UNIX like system

**Programming language**: Perl 5.10.0

**Other requirements**: BioPerl, R, BLAST, MAFFT, MOTHUR, MySQL 5.1, Apache, CGI, DBI.pm, DBD::mysql.pm

**License**: GNU General Public License.

**Any restrictions to use by non-academics**: license needed.

## Competing interests

The authors declare that they have no competing interests.

## Authors' contributions

JB and CS designed the study. RVP analyzed and wrote the code. RVP designed and developed the database and web interface. RVP wrote the draft of the manuscript and VN, CS, JB and RVP revised it. All authors read and approved the final manuscript.
